# Myelin oligodendrocyte glycoprotein antibody-associated disease (MOGAD) and Human Immunodeficiency virus infection: dilemmas in diagnosis and management: a case series

**DOI:** 10.1186/s13256-023-04191-7

**Published:** 2023-10-17

**Authors:** Yohane Gadama, Marié Du Preez, Jonathan Carr, Sarel Theron, Christine Albertyn, Kenneth Ssebambulidde, Deanna Saylor, Naeem Brey, Franclo Henning

**Affiliations:** 1 Division of Neurology, Department of Medicine, Faculty of Medicine and Health Sciences, Stellenbosch University, Cape Town, South Africa; 2 Malawi-Liverpool-Wellcome Trust Clinical Research Programme, Kamuzu University of Health Sciences, Blantyre, Malawi; 3 Department of Neurology, Johns Hopkins University School of Medicine, Baltimore, MD USA; 4 Research Department, Infectious Diseases Institute, Makerere University, Kampala, Uganda

**Keywords:** Myelin oligodendrocyte glycoprotein (MOG), MOGAD, Opportunistic infections, Advanced HIV disease, Aquaporin-4, Autoimmune diseases, Case report

## Abstract

**Background:**

Myelin oligodendrocyte glycoprotein antibody-associated disease (MOGAD) is a recently described autoimmune inflammatory disorder of the central nervous system (CNS). There is limited data on the association between Human Immunodeficiency virus (HIV) infection and MOGAD. We report three patients with HIV infection and myelin oligodendrocyte glycoprotein (MOG) antibodies in the setting of other central nervous system infections.

**Case descriptions:**

The first patient, a 44-year-old black African man, presented with acute disseminated encephalomyelitis (ADEM) with positive serum MOG antibodies. He made a significant recovery with corticosteroids but had a quick relapse and died from sepsis. The second patient, an 18-year-old black woman, presented with paraplegia and imaging revealed a longitudinally extensive transverse myelitis and had positive serum MOG antibodies. She remained paraplegic after methylprednisone and plasmapheresis treatments. Her rehabilitation was complicated by development of pulmonary embolism and tuberculosis. The third patient, a 43-year-old mixed-race woman, presented with bilateral painless visual loss. Her investigations were notable for positive MOG antibodies, positive Varicella Zoster Virus on cerebral spinal fluid (CSF) and hyperintense optic nerves on magnetic resonance imaging (MRI). Her vision did not improve with immunosuppression and eventually died from sepsis.

**Conclusion:**

Our cases illustrate the diagnostic and management challenges of MOGAD in the setting of advanced HIV infection, where the risk of CNS opportunistic infections is high even without the use of immunosuppression. The atypical clinical progression and the dilemmas in the diagnosis and treatment of these cases highlight gaps in the current knowledge of MOGAD among people with HIV that need further exploration.

## Background

Myelin oligodendrocyte glycoprotein antibody-associated disease (MOGAD) is a recently described autoimmune inflammatory disorder of the central nervous system (CNS) [1, 2]. Myelin oligodendrocyte glycoprotein (MOG) auto-antibodies induce an immune-mediated demyelination with a predilection for spinal cord, optic nerves, and the brain [3, 4]. Patients typically present with optic neuritis (bilateral or unilateral), transverse myelitis, acute disseminated encephalomyelitis (ADEM) and focal cortical disease [5, 6]. Clinically, there are distinct clinical and radiological features which distinguish MOGAD from multiple sclerosis and from aquaporin-4-seropositive neuromyelitis optica spectrum disorder (AQP4 + NMOSD) that have led to MOGAD being treated as a distinct disease [2, 3]. MOGAD is rarely associated with coexisting systemic autoimmune disorders [7], and infections are increasingly recognised as a significant trigger of MOGAD [8–11].

Globally, MOGAD is an uncommon condition, with recent reports indicating a prevalence of approximately 1.3–2.5 / 100,000, and annual incidence of approximately 3.4–4.8 / million [12]. There is no data on the association of HIV with MOGAD, and whether clinical presentation and outcomes of MOGAD in people with HIV are similar to the general population is unclear.

In this paper, we report three cases of ADEM, transverse myelitis and bilateral optic neuritis in people with HIV, all of whom had MOG antibodies. However, other clinical and laboratory findings made the diagnosis of MOGAD not straightforward. Through these cases, we discuss some of the challenges in diagnosing and managing MOGAD in patients with HIV and highlight gaps in our understanding of the pathophysiology of MOGAD that needs further elucidation.

## Case descriptions

### Case 1

A 44-year-old black man presented with a 2-day history of acute paraparesis with urinary incontinence and constipation. Two weeks prior to this admission, he had a generalized tonic–clonic seizure and was taken to his local clinic where he tested positive for HIV with a CD4 count of 18 cells/µL.

**Fig. 1:**
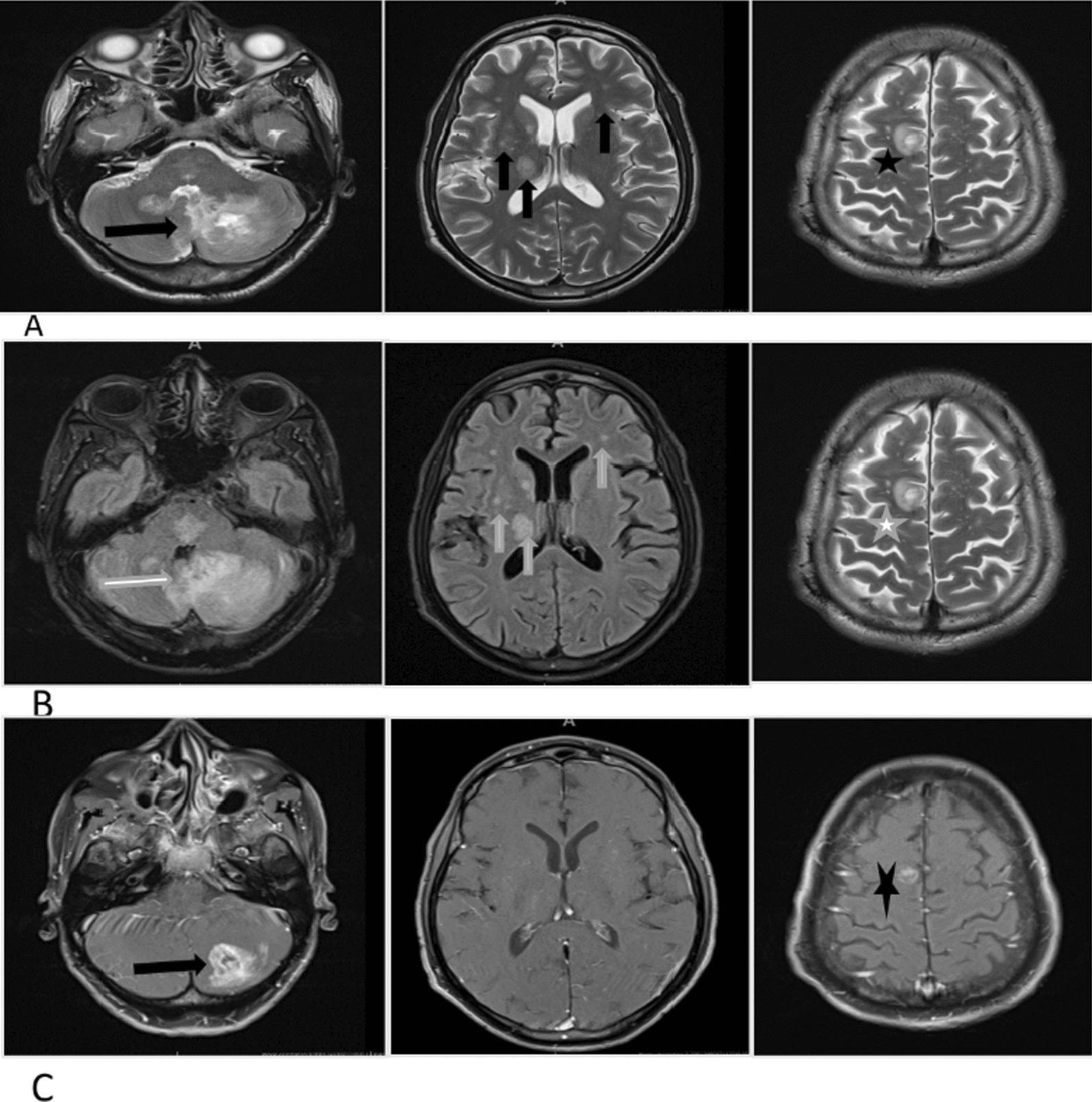
Pre-and post-contrast brain magnetic resonance imaging of case 1. Panel **A** (T2 weighted axial images): multiple bilateral hyperintensities demonstrated in the white and gray matter, with involvement of bilateral white mater lesions, the basal ganglia, thalami (black vertical arrows). A round subcortical right high parietal lesion with central hyperintensity and hypointense rim (black star). There is also a larger poorly defined lesion in the left cerebellum, with heterogenous signal intensity (black horizontal arrow). Panel **B** (Fluid attenuated inversion recovery axial images): multiple bilateral hyperintensities demonstrated in the white and gray matter, with involvement of bilateral white mater lesions, the basal ganglia, thalami (white vertical lines). A round subcortical right high parietal lesion with central hyperintensity and hypointense rim (white star). There is also a larger poorly defined lesion in the left cerebellum, with heterogenous signal intensity (white horizontal arrow). Panel **C** (T1 post-contrast axial images): an irregular peripheral enhancement of a subcortical right high parietal lesion (black star) and a prominent heterogenous enhancement with central non-enhancing foci of a lesion in the left cerebellum (black horizontal arrow)

**Fig. 2:**
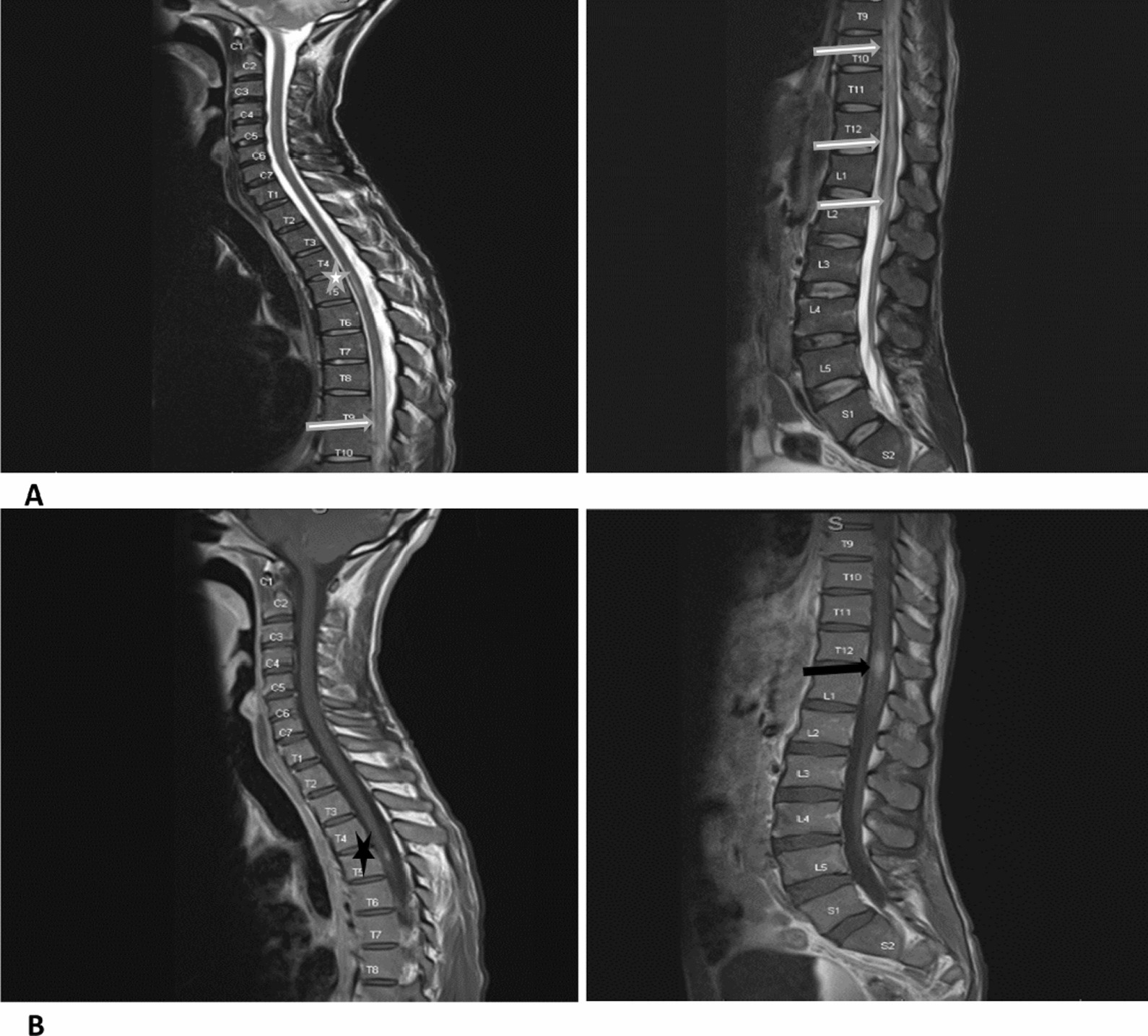
Whole spine magnetic resonance imaging of case 1. Panel **A**: T2 weighted sagittal images of whole spine demonstrating cord hyperintensities—one short segment at T5 (white star) and a long segment extending from T7 up to and involving the conus medullaris (white horizontal arrows). Panel **B**: T1 post-contrast sagittal images of whole spine showing small areas of intramedullary enhancement demonstrated at T5 (black star) and T12 (black horizontal arrow)

On clinical examination, he was generally wasted and had bilateral horizontal gaze-evoked nystagmus, bilateral upper limb ataxia, paraparesis, and a sensory level at T10. MRI of the brain showed multiple hyperintensities involving the bilateral white and grey matter (Fig. 1), and spine MRI revealed both long and short segments of increased signal on T2-weighted images with patchy areas of enhancement (Fig. 2).

Extensive blood tests were unremarkable, and active tuberculosis was not identified (Table 1). Toxoplasma serology suggested prior quiescent infection. Serum MOG antibodies were positive at 1:100 titer, while aquaporin-4 (AQP4) antibodies were negative. MOG antibodies were analyzed using a commercially available (Euroimmun, Lübeck, Germany) cell-based assay [13]. Cerebrospinal fluid analysis (CSF) revealed a raised protein and lymphocytic pleocytosis while infectious studies were unrevealing (Table 1).

**Table 1:** Laboratory findings of the three cases

Variable	Patient values on admission			Reference range*
	Case 1	Case 2	Case 3	
	*Blood*			
White cell count (× 10^9^/L)	11.3	4.54	5.15	3.92–10.40
Haemoglobin (g/dL)	14.2	12.2	11.5	13.0–17.0
Mean corpuscular volume (fL)	90.3	88.5	90.4	83.1–101.6
Platelets (× 10^9^/L)	264	248	128	171–388
Sodium (mmol/L)	132	139	140	136–145
Potassium (mmol/L)	4.6	4.3	3.5	3.5–5.1
Urea (mmol/L)	2.0	3.4	4.0	2.1–7.1
Creatinine (µmol/L)	46	63	55	64–104
Calcium (mmol/L)	2.31	2.09	2.25	2.15–2.50
Magnesium (mmol/L)	0.76	0.82	0.59	0.63–1.05
Inorganic phosphate (mmol/L))	1.12	1.35	0.99	0.78–1.42
Albumin (g/L)	38	34	38	35–52
C-Reactive Protein (mg/L)	12	4		< 10
Vitamin B12 (pmol/L)	778	284	367	145–569
TSH (mIU/L)	1.63	Not done		0.27–4.20
Cryptococcal Antigen (LFA)	Negative	Not done	Negative	
Treponema pallidum antibodies	Non-reactive	Non-reactive	Non-reactive	
HIV test (ELISA)	Positive	Positive	Positive	
Absolute CD4 count (cells/µL)	18	334	2	332—1642
AQP4* IgG antibodies	Negative	Negative	Negative	
MOG IgG antibodies*	Positive (1:100)	Positive (1:10)	Positive (1:10)	
*Toxoplasma gondii* serology	Done	Not done	Negative	
IgG	Positive			
IgM	Negative			
Avidity index	95%			
Hepatitis B & C	Negative	Negative		Negative
Anti-nuclear antibodies	Negative	Not done	Negative	
Anti-double strand DNA antibodies	–	–	Negative	
Rheumatoid factor IU/mL	–	Negative	< 10	< 14
Antiphospholipid antibodies	–	–	Negative	
Anti-SS-A (Ro) & Anti-SS-B (La) antibodies	–	–	Negative	
	*Cerebrospinal fluid*			
Glucose (mmol/L)	Not done	2.2 (serum 4.9)	6.4	2.7–4.4
Protein (g/L)	0.89	1.93	0.39	0.15–0.45
WBC (cells/µL)				< 5
Polymorphs (cells/µL)	0	6	0	
Lymphocytes (cells/µL)	16	13	0	
Erythrocytes (cells/µL)	0	4	0	
IgG index	–	0.94	–	
Gram stain	No organisms	No organisms		
Viral panel	Negative	Negative	IgG positive for Epstein-barr virus and Varicella Zoster virus) – Viral load not available	
Cryptococcal antigen test	Negative	Negative	Negative	
Treponema antibodies	negative	Negative	Negative	
Xpert MTB/Rif	MTB not detected	MTB not detected	MTB not detected	
	*Sputum*			
Xpert MTB/Rif ultra	Not detected	Not detected	Not detected	
Auranine O stain	Negative	–	Negative	
MTB culture (after 35 days)	Negative	Negative	Negative	

*Serum MOG and aquaporin-4 autoantibodies were analyzed using a commercially available cell based assay (Euroimmun, Lübeck, Germany) [8]

The ADEM presentation, MOG antibodies and absence of an identified active infection were consistent with a diagnosis of MOGAD [2]. Treatment of methylprednisolone 1 g/day intravenously for five days was initiated, followed by oral prednisone 1 mg/kg. By the second week of admission, nystagmus and ataxia had resolved, and he was mobilizing without support. He was discharged three weeks later but was lost to follow-up until he was re-admitted eight weeks later with recurrent paraplegia (MRC power grading of 0/5, sensory level at T6 and normal upper limbs, coordination, and cognition). He had not initiated antiretrovirals (ARVs) and was still taking prednisone 50 mg daily. This second admission was further complicated by hospital-acquired pneumonia and a peri-anal abscess. Despite appropriate antimicrobial treatment, he died a week after re-admission.

### Case 2

**Fig. 3:**
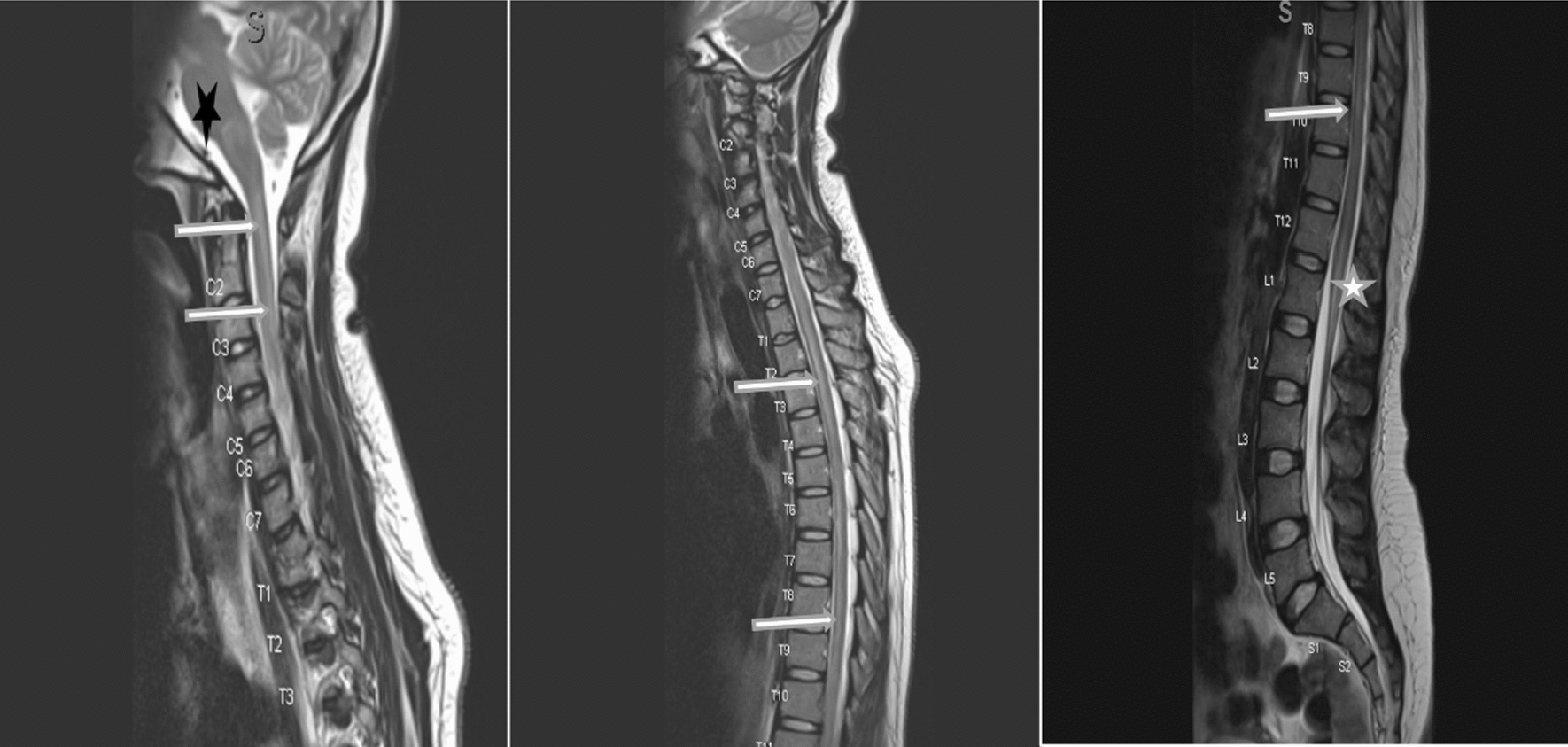
Whole spine magnetic resonance imaging of case 2. T2 weighted images showing hyperintensity of the whole spinal cord (white horizontal arrows), with involvement of the brainstem (black star) and conus medularis (white star)

An 18-year-old black woman presented with bilateral lower limb weakness and urinary retention that was discovered upon awakening followed by dysphagia and dysarthria the next day. On examination, she was in respiratory distress and paraplegic with a sensory level at T4. The upper limb and cranial nerve examinations were normal as were routine hematology and biochemistry investigations (Table 1). However, she tested positive for HIV with a CD4 count of 334 cells/μL. CSF analysis revealed a raised protein and lymphocytic pleocytosis, but no infection was identified. Additional tests for tuberculosis were also negative. Serum MOG antibodies were positive (titer 1:10). MRI revealed a longitudinally extensive non-enhancing transverse myelitis (LETM) involving the entire spinal cord, as well as T2 hyperintense signal change within the medulla (Fig. 3). The remainder of the brain MRI was normal.

The presence of LETM with conus medullaris involvement and MOG antibodies and the absence of an alternative etiology for the clinical presentation was suggestive of MOGAD [2]. She was intubated and admitted to the intensive care unit (ICU) and treated with intravenous methylprednisolone 1 g/day for 4 days. However, the weakness progressed to involve the upper limbs and she was treated with five sessions of plasma exchange over ten days, and oral prednisone at 1 mg/kg/day was continued thereafter. She was ultimately discharged to a rehabilitation hospital with no bulbar weakness or respiratory compromise, normal upper limb strength, but unchanged paraplegia (MRC power grading 1/5). Six weeks post-discharge, she developed a deep venous thrombosis and subsequent pulmonary thromboembolism. She was re-admitted to the ICU and was anticoagulated with warfarin, and re-testing for TB revealed positive urine lipoarabinomannan (LAM) and sputum GeneXpert MTB/Rif. A repeat testing for MOG antibodies remained positive (titer 1:10) and AQP4 was negative. She was appropriately treated for the TB and pulmonary thromboembolism and discharged back to the rehabilitation facility. On follow-up three months later, while on TB treatment and ARVs, she was still paraplegic and confined to a wheelchair.

### Case 3

A 43-year-old mixed-race female with no known comorbidities presented with a two-day history of sudden onset, painless, bilateral visual loss. She had no history of preceding viral illnesses, no limb weakness, and no bulbar, bowel or bladder dysfunction. General examination revealed a vesicular rash over her left breast and normal vital signs. She had no light perception bilaterally and dilated pupils which were sluggishly reactive to light. Fundoscopy revealed blurred nasal margins of the optic discs and no papilledema. Eye movements and the remainder of the neurological evaluation was normal.

**Fig. 4:**
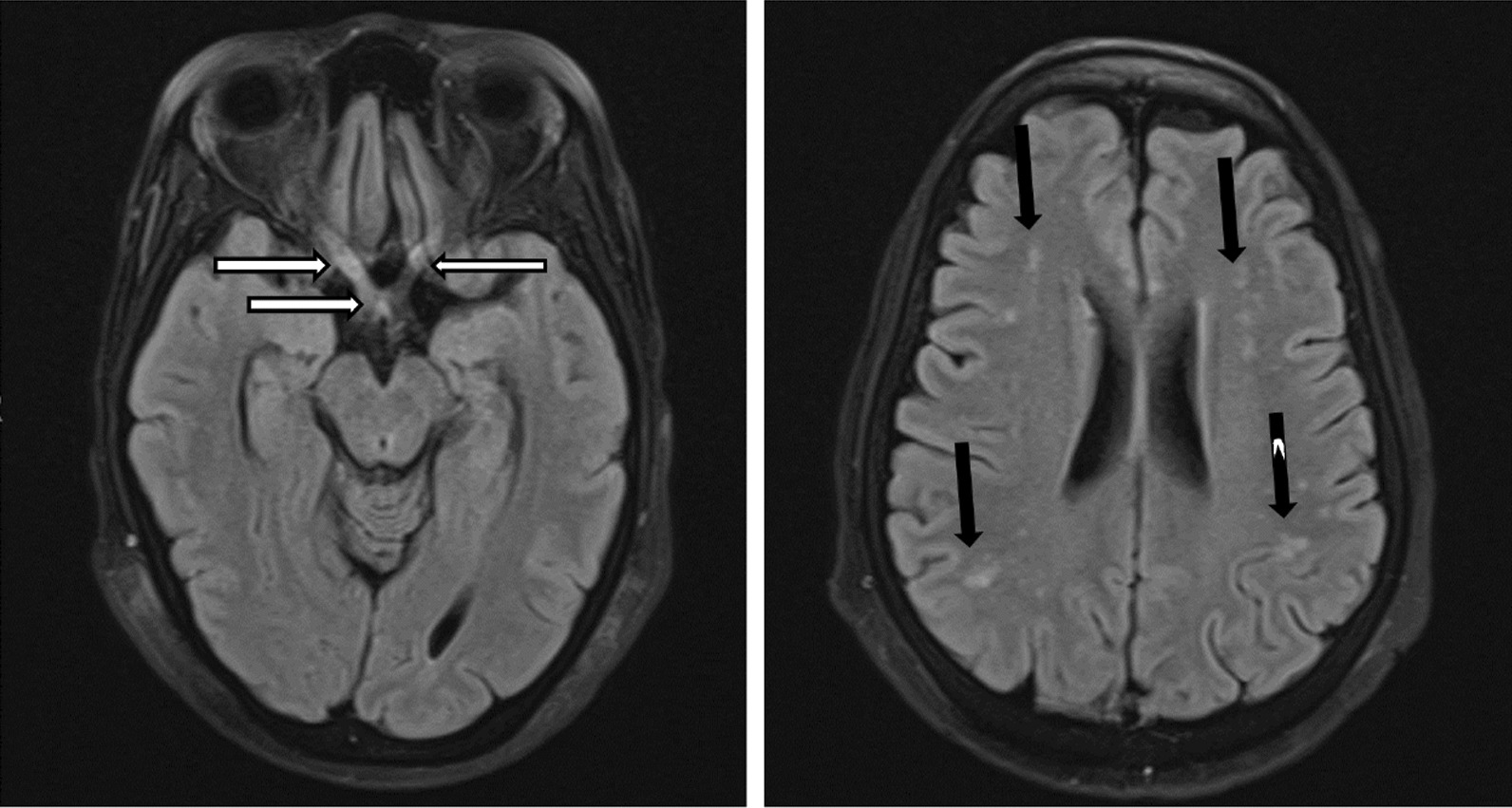
Brain magnetic resonance imaging of case 3. (Fluid attenuated inversion recovery axial images sequence)—bilateral hyperintensity of intra-orbital and intracranial optic nerves (R > L) as well as optic chiasm and right optic tract (horizontal white arrows). Bilateral normal orbital globes with no collections or masses. Generalized cerebral atrophy. Multiple bilateral scattered hyperintensities (vertical black arrows), no contrast enhancement when gadolinium was administered

MRI of the brain (Fig. 4) demonstrated T2 and FLAIR hyperintensity of both optic nerves extending to the optic chiasm, with no contrast enhancement. Laboratory investigations (Table 1) were notable for a positive HIV test with a CD4 count of 2 cells/µL and the presence of MOG antibodies (titre 1:10).

Her clinical presentation, positive MOG antibodies and supporting radiological findings supported the diagnosis of MOGAD [2]. She was pulsed with methylprednisone 1 g/daily for five days with no response, and subsequently received five cycles of plasma exchange, again with no improvement in vision. The presence of varicella zoster virus (VZV) IgG in CSF and poor response to corticosteroids and plasma exchange was atypical for MOGAD. Although unusual, we considered VZV as a possible cause of the optic neuritis and added intravenous acyclovir, 500 mg 8 hourly, to her treatment. While on day six of antiviral treatment, she developed delirium, and work-up revealed ertapenem-susceptible *Klebsiella pneumoniae* on urine culture. She also developed acute kidney injury, and the ertapenem dose was adjusted accordingly. Despite correction of renal impairment and appropriate antimicrobial treatment, she died on day twenty-four of hospitalisation.

## Discussion

There is limited data on the association between MOGAD and HIV infection, with two case reports found in literature. Myelopathy with positive serum MOG antibodies was present in both of these cases, but one had a well-controlled HIV infection [14] while the other was seroconverting at the time of presentation [15]. The three patients reported here had aggressive presentations: the first with an ADEM presentation, the second with LETM extending from the brainstem to conus medullaris with respiratory compromise, and the third with bilateral optic neuritis that did not improve after immunosuppression and plasma exchange. All patients were antiretroviral therapy (ART)-naïve, and cases one and three had advanced HIV infection, as evidenced by low CD4 counts. According to existing understanding of MOGAD, our cases are unique due to their poor outcomes despite appropriate treatments and associations with HIV and, possibly, opportunistic infections. Although diagnostic work-up for infections that would explain the clinical presentations was negative, both infectious causes and MOGAD, based on consensus diagnostic criteria [2], seemed plausible in all three cases.

Case 1 showed significant improvement initially and was able to ambulate after admission, consistent with prior literature suggesting that ADEM presentations of MOGAD are more commonly monophasic with a good prognosis and infrequent relapses [5, 6, 16]. However, our patient relapsed in less than two months and died from sepsis. This is unusual for MOGAD, especially if corticosteroids are continued after an initial attack, and transverse myelitis was present at onset [17, 18]. This raises the possibility that an unidentified infection that could cause a similar presentation and may also have initially improved with steroids, such as tuberculosis (TB), could have contributed to the presentation. Moreover, the impact of infections on severity and relapse risk of MOGAD is unknown [1]. Case 2 showed resolution of respiratory compromise and upper limb weakness with plasma exchange but remained paraplegic. While we considered TB during her first admission, the negative microbiological testing and imaging findings (which were more suggestive of MOGAD) argued against the diagnosis. Whether she developed TB subsequent to further corticosteroid-immunosuppression therapy or had two disease processes (MOGAD and TB) at the time of her initial presentation is difficult to ascertain. For instance, initial CSF findings (raised protein, lymphocytic pleocytosis and low glucose) supported a diagnosis of CNS TB, although the same can be seen in MOGAD, and current TB diagnostics are notorious for their undesirably low sensitivity, making a TB diagnosis difficult to confirm [19, 20]. Case 3 had bilateral optic neuritis that also did not improve after intravenous steroids and plasma exchange. It is possible that the optic neuritis may have been caused by VZV infection, as evidenced by the absence of pain, presence of a vesicular rash and positive VZV IgG in CSF [21]. In addition, while it is uncommon for MOGAD-related optic neuritis to not improve after immunotherapy, a poor treatment response is not uncommon in VZV [21]. The presence of MOG antibodies in patients with VZV infection is rare [22] and bilateral optic neuritis from VZV is uncommon [21]. Systematic testing of MOG antibodies in people with VZV optic neuritis is likely not commonly completed and the presence of the two in our patient made a definitive diagnosis of either VZV or MOGAD optic neuritis challenging.

These atypical presentations of MOGAD in patients with significant immunocompromise raises the possibility of immune dysregulation from HIV infection [23, 24]. Moreover, atypical presentations of both ADEM and NMOSD in HIV positive patients have been reported and immune dysregulation is thought to play a role [23–25]. Although speculative, MOGAD may have been a possible explanation for those ADEM or NMOSD-seronegative cases with atypical presentations prior to widespread availability of MOG antibody testing. The pathophysiology of MOGAD is not completely understood; however, there are hypotheses that complement activation and CD4-positive T cell inflammation (unlike CD8 T cells in multiple sclerosis) predominate its pathogenesis [26]. The presence of MOG antibodies and low CD4 counts in patients with advanced HIV challenges some of the postulated theories of the MOGAD pathogenesis. Thus, studying MOGAD in HIV infected patients may provide clues as to the type of immune dysregulation occurring in MOGAD patients and hence contribute to further understanding of its pathophysiology.

Given the potential of false positives, careful clinical reasoning is recommended and the recently proposed diagnostic criteria for MOGAD may be useful in clinical practice [2]. The low positive titres and unsatisfactory improvement after immunosuppression in our cases may likely represent a ‘bystander effect’- mechanism where the ongoing immune response to infection and accompanying inflammation causes normally hidden auto-antigens to become exposed to the immunological response [27]. Although the pathogenicity of MOG antibodies and their utility in prognostication of MOGAD is unresolved, it is becoming evident that the positive predictive value (PPV) of MOG antibodies is higher with clear positive MOG antibodies (PPV of 100% for titer 1:1000, 82% for titre 1:100 and 51% for low positive titres < 1:40) [1, 28]. In light of these findings, interpreting low-positive titres in patients presenting with a clinical phenotype of MOGAD will continue to be challenging, particularly in patients with highly suspected but unidentified infections. Thus, in patients with a clinical presentation suggestive of MOGAD and with immunosuppression, MOG seropositivity (especially at low titers) should be interpreted cautiously and a thorough work-up for alternative aetiologies (like infections) should be completed.

When managing autoimmune disorders in immunosuppressed patients, balancing the benefits of (long-term) immunosuppression with the increased risk of infections, particularly opportunistic infections such as tuberculosis, is an additional important consideration [29, 30]. Indeed, two of our patients re-presented with infections. Unfortunately, there are no clear guidelines on how to approach this clinical conundrum; nonetheless, it may be prudent to initiate isoniazid preventative therapy prior to long-term immunosuppression.

Further limitations in the management of our patients include the absence of histopathological investigations that could have helped to further understand the disease process underlying the clinical presentations. Additionally, we could not obtain repeat imaging and follow-up MOG antibody testing, as two of our patients died during hospitalization.

## Conclusion

In conclusion, these cases reveal gaps in our understanding of MOGAD, and in particular to the interpretation of MOG antibodies in people with HIV and illustrate problematic scenarios that clinicians working in HIV medicine may encounter. With the increasing availability of MOG antibody testing, clinicians practising in regions with a high prevalence of CNS opportunistic infections that may mimic MOGAD should be aware of the limitations of the test and interpret results with caution. Further studies investigating a possible association between HIV infection and MOGAD, and the role of immune dysregulation in the pathophysiology of MOGAD, are warranted. There is also a need for prospective and long-term follow-up data for a better understanding of the epidemiology, clinical presentation and outcomes of MOGAD among HIV-infected patients.

## Data Availability

Not applicable.

## Abbreviations

ADEM: Acute disseminated encephalomyelitis
MOG: Myelin oligodendrocyte glycoprotein
MOGAD: Myelin oligodendrocyte glycoprotein antibody-associated diseased
CNS: Central nervous system
LETM: Longitudinally extensive transverse myelitis
HIV: Human immunodeficiency virus
NMOSD: Neuromyelitis optica-spectrum disorder
VZV: Varicella zoster virus
PPV: Positive predictive value
